# Metastatic prostatic adenocarcinoma presenting as generalized lymphadenopathy unmasked by a COVID booster vaccine

**DOI:** 10.1002/ccr3.8278

**Published:** 2023-11-28

**Authors:** Kavya Bharathidasan, Vivie Tran, Sayed Reshad Ghafouri, Shabnam Rehman, Luis Brandi

**Affiliations:** ^1^ Department of Internal Medicine Texas Tech University Health Science Center Lubbock Texas USA; ^2^ Department of Internal Medicine, Division of Hematology/Oncology Texas Tech University Health Science Center Lubbock Texas USA; ^3^ Department of Pathology Texas Tech University Health Science Center Lubbock Texas USA

**Keywords:** COVID vaccine, generalized lymphadenopathy, immunization reaction, metastatic prostate cancer, prostate adenocarcinoma

## Abstract

**Key Clinical Message:**

Lymphadenopathy following recent immunization is usually regional. Generalized lymphadenopathy should arouse suspicion for alternative underlying pathology. Prostate adenocarcinoma should be considered in the differential diagnosis for malignancy in an elderly male patient. Metastatic prostate adenocarcinoma can have good prognostic outcomes if treatment is started promptly, even in the setting of widespread disease.

**Abstract:**

Generalized lymphadenopathy is commonly attributed to infectious causes or malignancy, often lymphoproliferative disorders. We present a rare case of metastatic prostate cancer diagnosed after initially presenting as generalized lymphadenopathy following a coronavirus disease 2019 (COVID) booster vaccination. A 70‐year‐old Hispanic male presented with left lower quadrant abdominal pain, nausea, headache, myalgia, severe constipation, and a right‐sided neck swelling that had been increasing in size since the day of his vaccination. Computed tomography (CT) scans of soft tissue neck, chest, abdomen, and pelvis with contrast showed extensive lymphadenopathy. Ultrasound‐guided biopsy results of the enlarged right supraclavicular node and prostate revealed histopathology consistent with that of prostate acinar adenocarcinoma. He started on bicalutamide for 4 weeks, transitioned to gonadotropin releasing hormone analogue (leuprolide) injections every 3 months and oral androgen receptor signaling inhibitor (abiraterone with prednisone daily). PSA level declined from 121 ng/mL at diagnosis to 1.3 ng/mL after 3 months of therapy, and repeat imaging showed marked improvement in the size of his mediastinal, retroperitoneal, and pelvic lymphadenopathy. To the best of our knowledge, this is the first case reported of a COVID vaccine booster uncovering lymphadenopathy leading to the diagnosis of metastatic prostate cancer.

## Graphical abstract

Large right‐sided supraclavicular lymphadenopathy observed in a 70‐year‐old male patient following COVID booster vaccination.

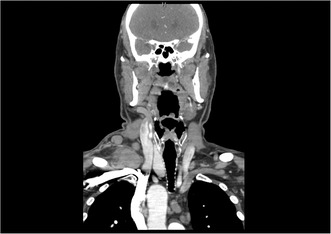

## 1 INTRODUCTION

Generalized lymphadenopathy is commonly attributed to infectious causes such as human immunodeficiency virus (HIV) infection, Epstein–Barr virus (EBV), cytomegalovirus (CMV), herpes simplex virus (HSV), human herpesvirus 6 (HHV‐6), tuberculosis (TB), syphilis, cat‐scratch disease caused by Bartonella henselae, or other parasitic and fungal infections. Lymphadenopathy can also occur following recent immunization and is usually transient requiring no further workup. Ho et al described that less than 1.1% of individuals developed lymphadenopathy following coronavirus disease 2019 (COVID‐19) vaccination lasting on average between 10 days and 2 months. It was often identified incidentally on computed tomography (CT) scans, routine screening mammography, or positron emission tomography scans, breast MRI, and ultrasounds. The most common site for COVID‐19 vaccine‐associated lymphadenopathy is usually axillary followed by supraclavicular and cervical lymphadenopathy.^1^ In the following report, we present a rare case of metastatic prostate cancer diagnosed after initially presenting as generalized lymphadenopathy following a COVID booster vaccination.

## 2 CASE DESCRIPTION

**FIGURE 1:**
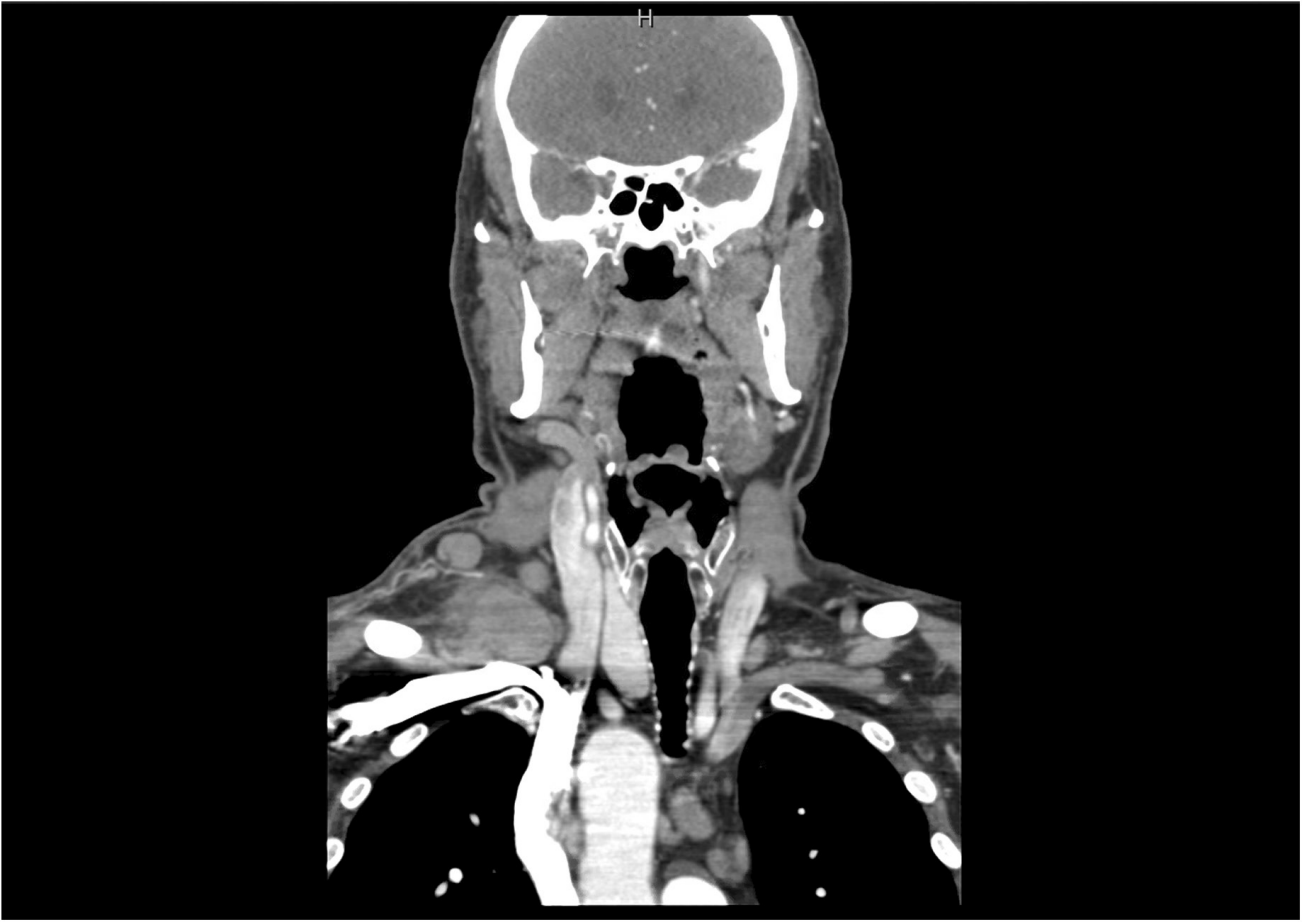
CT soft tissue neck with contrast (transverse view) highlighting large right sided supraclavicular lymphadenopathy.

We present the case of a 70‐year‐old Hispanic male who presented to the urgent care clinic with left lower quadrant abdominal pain, nausea, headache, myalgia, and severe constipation. He had just received his fourth COVID booster vaccine the day prior and stated to have been asymptomatic prior to receiving the immunization. His only past medical history is well‐controlled benign essential hypertension for which he takes amlodipine 10 mg daily. The patient has been adherent to his regular annual wellness visits with his family medicine primary care physician. He is a past‐smoker (40 pack year history) and quit 4 years prior to presentation when he was noted to have an incidental stable descending thoracic aortic aneurysm of 4.2 cm × 4.4 cm noted on chest CT. He had completed all age‐appropriate cancer screenings including negative low‐dose CT for lung cancer screening a year prior which did not show any adenopathy. His prostate specific antigen was noted to be elevated in 2016 at 5.0 ng/mL (0.1–4.0 ng/mL) but was not followed up as the patient denied urinary symptoms. An abdominal x‐ray showed no obvious bowel obstruction or extravasation of air; hence the patient was reassured to be experiencing the expected side effects of the vaccine and was sent home. He returned to the emergency room 2 days later complaining of a right‐sided neck swelling that had been increasing in size since the day of his vaccination. A CT soft tissue neck with contrast (Figures 1,, 2) showed bilateral posterior triangle/supraclavicular adenopathy, most extensive on the right with a large nodal mass measuring up to 4.7 × 2.4 × 3.2 cm with surrounding inflammatory changes. There was also bulky mediastinal adenopathy with the largest node being a right suprahilar lymph node measuring up to 5 cm in maximal diameter. A contrast CT scan of the chest, abdomen, and pelvis (Figure 3) showed a mass interposed between the superior vena cava and the right side of the ascending aorta measuring 4.2 × 5.9 × 4.5 cm, a portion interposed between the ascending and descending aorta displacing the trachea to the left side, enlarged level 5, and subcarinal level 7 lymphadenopathy, distal mediastinal tumor above the diaphragm with extension of the retroperitoneal tumor inferiorly along the aorta into the pelvis causing hydronephrosis and hydroureter of the left kidney. The tumor surrounded the inferior mesenteric artery and extended into the pelvis and into the mesentery in the presacral space. Pelvic tumor was also seen in the mesentery surrounding the sigmoid colon and in the sub‐peritoneal space adjacent to the enlarged prostate likely contributing to his constipation. Radiologically, the patient's findings were highly suspicious for a lymphoproliferative disorder, and the hematology/oncology team was consulted for the same. However, on examining the ultrasound‐guided biopsy results of the enlarged right supraclavicular node, the histopathology was consistent with that of prostate acinar adenocarcinoma (Figure 4). The negative staining pattern for CK7 and CK20 with patchy weak PSA antigen staining and positive prostate marker immunohistochemistry stain for NKX3A further supported the diagnosis. Prostate specific antigen (PSA) level was then checked which measured 121.3 ng/mL (reference range 0.1–4.0 ng/mL). The patient denied prior urinary symptoms apart from left flank/lower abdominal pain which began after receiving the COVID vaccine. A bone scan was performed, which suggested very early metastatic disease in the manubrium, inferior tip of right scapula, inferior aspect of right iliac wing, and T11 vertebral body. A subsequent prostate biopsy confirmed prostate adenocarcinoma with a Gleason score of 8 (4 + 4). He underwent left ureteral stent placement and was started on bicalutamide for 4 weeks prior to discharge from the hospital. After establishing in the outpatient oncology clinic, he was transitioned to gonadotropin releasing hormone analogue (leuprolide) injections every 3 months and oral androgen receptor signaling inhibitor (abiraterone with prednisone daily). PSA level declined to 1.3 ng/mL after 3 months of therapy, and repeat imaging showed marked improvement in the size of his mediastinal, retroperitoneal, and pelvic lymphadenopathy.

**FIGURE 2:**
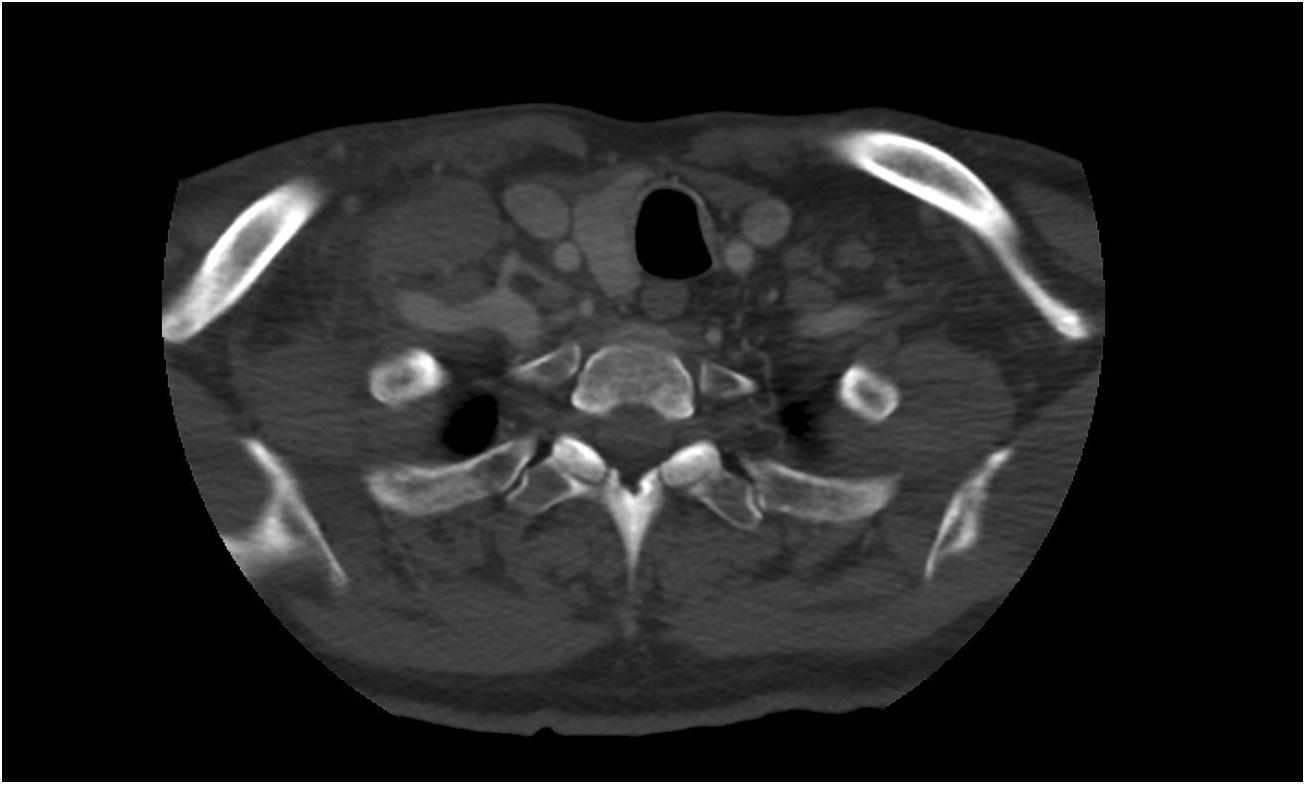
CT soft tissue neck with contrast (coronal view) highlighting large right sided supraclavicular lymphadenopathy.

**FIGURE 3:**
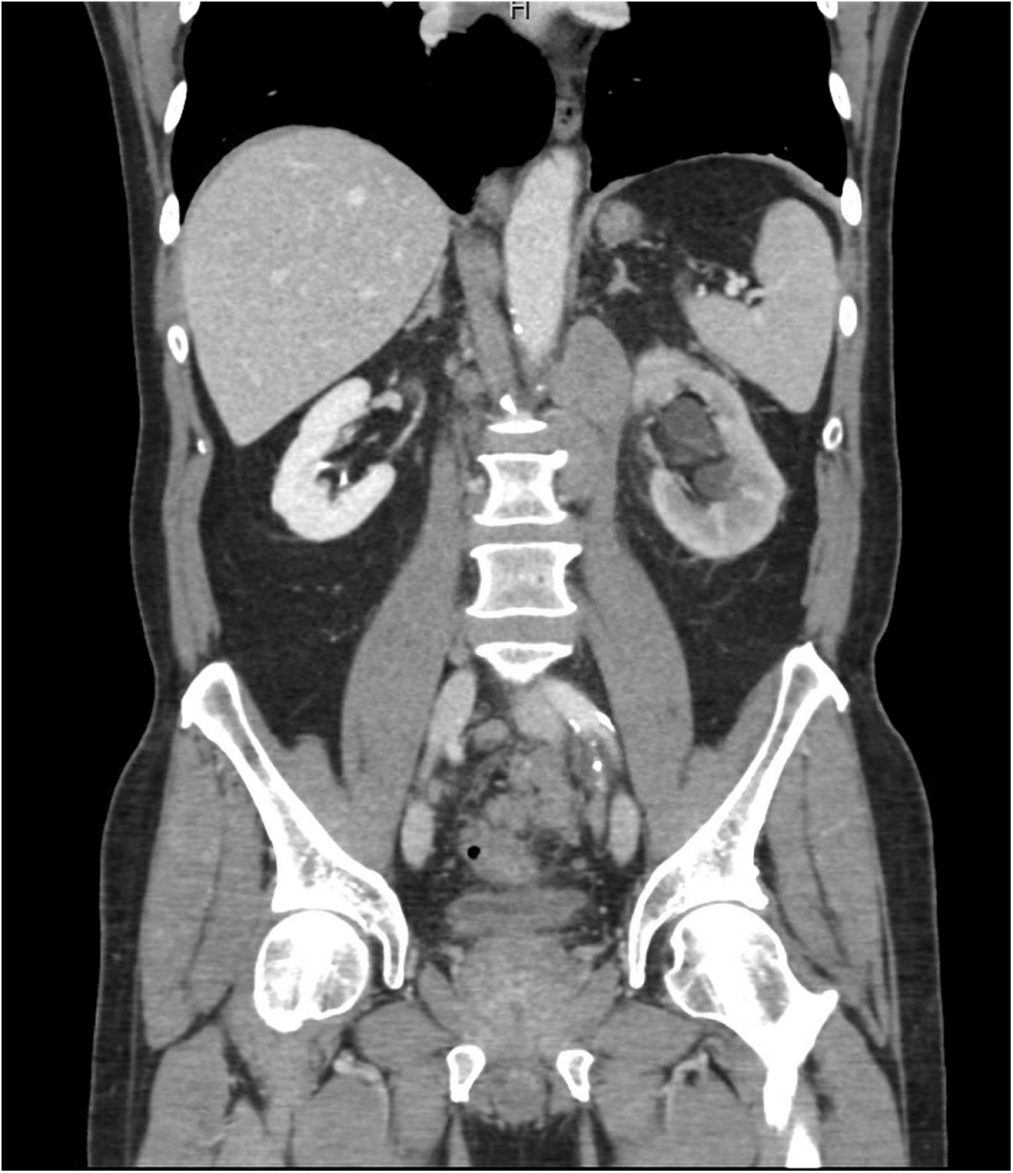
CT abdomen/pelvis with contrast (coronal view) showing obstructive retroperitoneal lymph node causing left sided hydronephrosis and hydroureter along with enlarged prostate.

**FIGURE 4:**
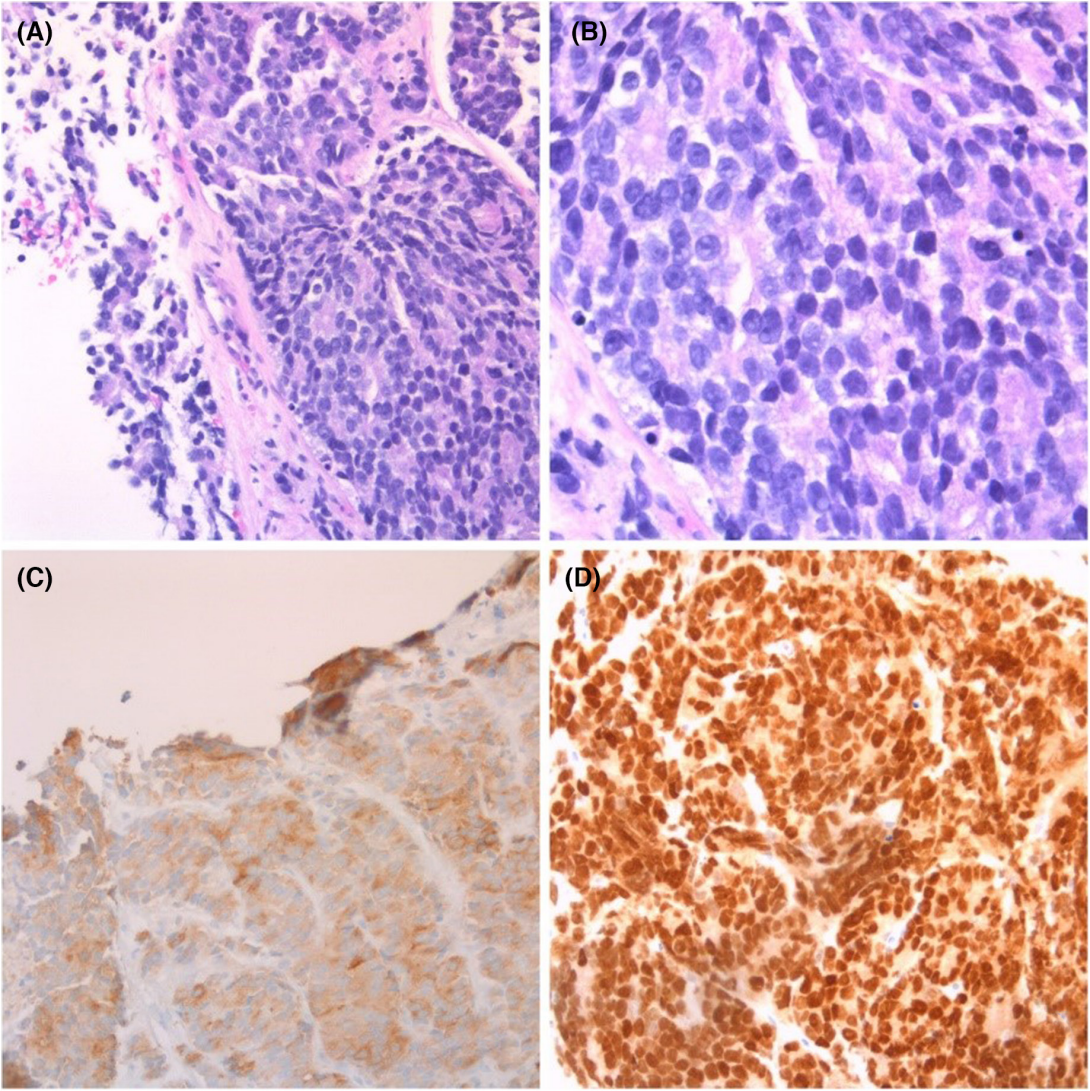
Histopathology slides. (A) Hematoxylin/eosin staining at 20x magnification showing prostatic acinar adenocarcinoma. (B) hematoxylin/eosin staining at 40x magnification. (C) patchy positive staining for prostate specific antigen (PSA). (D) positive prostate marker immunohistochemistry stain for NKX3A.

## 3 DISCUSSION

Generalized lymphadenopathy in an elderly male can have a wide differential diagnosis including, but not limited to, bacterial infections for example, cat scratch disease; viral infections for example, Hepatitis B, HIV; Mycobacterium TB; cancers for example, leukemia and lymphoma, lymphoproliferative disorders; fungal infections, autoimmune conditions for example, systemic lupus erythematosus, rheumatoid arthritis, dermatomyositis, or medications. In the United States, prostate cancer is the most diagnosed male malignancy and one of the leading causes of cancer‐related death. Approximately 12.9% of all men with be diagnosed with prostate cancer in their lifetime. At the time of diagnosis, 82% of prostate cancers are confined to the prostate and regional lymph nodes and have a 100% 5‐year survival rate. However, 8% of cases have already metastasized at the time of diagnosis and confer a 34.1% 5‐year survival rate.^2^ The most common sites of distant spread include bones (84%), distant lymph nodes (10.6%), liver (10.2%), and thorax (9.1%).^3^ Metastasis to cervical lymph nodes is rare, though becoming increasingly reported in recent literature.

Prostate cancer most commonly spreads to regional lymph nodes, pelvic organs via direct invasion, or to the axial skeleton. It uncommonly metastasizes to cervical lymph nodes. Patients usually present with urological symptoms before metastases become extensive. However, our patient presented to the hospital initially due to his enlarging right supraclavicular mass aggravated by a COVID booster vaccine. Generalized lymphadenopathy has been described following COVID vaccination, though most often confined to the axillary region. Imaging is not routinely recommended until 6 weeks postvaccination.^4^ It is likely that our patient had preexisting lymphadenopathy that he noticed only following immunization. To the best of our knowledge, this is the first case reported of a COVID vaccine booster uncovering lymphadenopathy leading to the diagnosis of metastatic prostate cancer. In a recent literature review, Liu et al note that 58 cases have been reported where cervical lymphadenopathy is the initial presentation of metastatic prostate cancer. It is interesting to note that only 7 of the 58 patients presented with right‐sided supraclavicular lymphadenopathy since more commonly, retrograde lymphatic spread via the left jugular trunk leads to the left‐sided cervical lymph nodes.^5^ Though we obtained biopsies only from the right supraclavicular node and prostate, the distribution of other enlarged lymph nodes and their decrease in size following androgen deprivation therapy, together with decline in PSA from 121 ng/mL to 1.3 ng/mL favors the assumption that the generalized lymphadenopathy was secondary to metastatic spread and not any other secondary or additional diagnosis. COVID‐19 vaccination has been noted to be safe and effective in the elderly with lower adverse events reported compared to younger populations. Injection site pain, fatigue, and headache are the most reported adverse effects. Rarely, thromboembolic phenomena have been reported with mRNA vaccines and viral vector vaccines.^6^ Interestingly, metastatic disease, advanced age, and male sex in patients with solid tumors are independent risk factors that have been shown to reduce antibody response following vaccination. Vaccine induced lymphadenopathy has been noted in patients with preexisting breast cancer and melanoma, with persistence of lymphadenopathy for more than 6 weeks in 29% of patients. However, the safety and efficacy profile of the vaccine are comparable to individuals without cancer, and vaccination has not been shown to worsen cancer outcomes by any means. It is important to distinguish vaccine induced lymphadenopathy from progression of cancer or cancer metastasis of patients already on therapy.^7^ Specifically with regards to genitourinary cancers, vaccination against COVID‐19 has shown to benefit patients by minimally interrupting treatment, causing lesser breakthrough infections with no significant vaccine related adverse effects.^8^

## 4 CONCLUSIONS

Our case highlights the importance of investigating a PSA level when malignancy is suspected in an elderly male patient regardless of the initial presenting tumor site or symptom. Though such extensive metastasis usually yields a poor prognosis, our patient had dramatic improvement with prompt initiation of treatment. The patient's generalized lymphadenopathy‐ particularly enlarged right supraclavicular lymph node, likely reactive secondary to COVID‐19 immunization, prompted further imaging and biopsy thereby leading us to his diagnosis of metastatic prostate adenocarcinoma which may have otherwise gone undetected until further disease progression.

## AUTHOR CONTRIBUTIONS

**Kavya Bharathidasan:** Writing – original draft; writing – review and editing. **Vivie Tran:** Writing – original draft; writing – review and editing. **Sayed Reshad Ghafouri:** Validation; writing – review and editing. **Shabnam Rehman:** Supervision; validation; visualization; writing – review and editing. **Luis Brandi:** Resources; visualization.

## FUNDING INFORMATION

None.

## CONFLICT OF INTEREST STATEMENT

The authors have no conflict of interest to declare.

## CONSENT

The authors have obtained written informed consent from the patient's medical power of attorney to publish his medical history/course and case details in accordance with the journal's patient consent policy.

## Data Availability

Data sharing is not applicable to this article as no new data were created or analyzed in this study.
